# Kilts and Physic

**Published:** 1887-04-23

**Authors:** 


### KILTS AND PHYSIC.

Everybody in Glasgow does not wear kilts, but almost everybody is a Scotchman or as sharp as a Scotchman. The hundred thousand *Irish*, of course, do not count. The Glasgow doctors are feeling the pinch of the times, and with the natural instinct of their countrymen are looking about to find the reason why. A true Scotchman thinks he ought to be able to live, and if he can't, he is bound to make somebody uncomfortable. Doctors have a right to exist, if they can; and especially Scotch doctors. At least, that would probably be considered incontrovertible on the banks of the Clyde. We heartily wish the Glasgow practitioners success in the difficult task they have set themselves. They have a hard nut to crack. London has tried a similar nut, and only succeeded in breaking its molar teeth. Liverpool and Manchester, to say nothing of Birmingham and Leicester, have each had an innings, and the scoring has made no headway at all against the bowling.

The point of the whole matter is this, that a great many of the people in Glasgow refuse to go to doctors who charge fees, because they can get what they want at certain hospitals for nothing. The cynic at once says, "What fools they would be to do otherwise." That is the gist of the whole matter in London and other large towns as well as in Glasgow. The question is rapidly reaching a *burning* stage. The pressure of the times is forcing it to the front. We do not know any place in which it is more likely to be fought out to a just issue than at Glasgow. London is too large, too inorganic, too inchoate to deal with any question of the kind. Glasgow, although the second or third city in the Empire, is only about an eighth of its size. It possesses, however, all the attributes of a capital city; a vast population; infinite variety of trades and manufactures; lawyers, painters, preachers, and physicians; a famous university,—a port, a poorhouse, and a prison; with all kinds of public institutions, great and small. It is *one* city, not many, as London is. It has one leading class, one middle class, and one poor class; and these classes so overlap and interweave, that everybody has some kind of connection with every class, at least to the extent of knowing something about it; and this distinctive unity of the city, coupled with its manifold diversity, gives it advantages in dealing with the question at issue, such as are possessed by very few large centres of population.

We have great respect for the practical brains of Scotchmen, and it is in no spirit of self-confidence that we venture to offer them a few counsels. The first thing they have to do is to make up their minds to carry the business to a definite conclusion. Most of them know the reward of him who puts his hand to the plough and then looks back. *Non possumus* is practically the cry in London, and it is an unmanly and contemptible utterance for grown-up men. Instead of saying "we cannot," the Glasgow people should say "we can and will." It is scandalous and pitiful that respectable tradesmen and well-paid artisans should buy their food, their clothing, their theology, and their amusements at a just price, and pay for them; and whenever they are ill go sponging on the nearest hospital, or whining for subscribers' cards. Nowhere is good hospital accommodation more abundant than in Scotland, and nowhere is the interpretation of the suitability of persons more liberal. But every charity has its limits; and there must be limits to the operation of medical charities. By all means, therefore, let the Glasgow public men and doctors set their faces like flints, and probe the evil to the very bottom.

We would say also to them—make sure of your facts. In London and elsewhere this has been the rock on which reformers have generally split. The advocates of change have exaggerated the extent of the evil, and so laid themselves open to the most damaging criticisms on the part of those who see nothing but excellence in things as they are. There should be no difficulty in finding out all that it is necessary or desirable to know. It is not for us to make suggestions about details, but it will be easy to obtain information from the London hospitals, and from Manchester, Liverpool, Leicester, and other places as to what has been done by those who have already had experience in similar work.

Our final counsel is this: when the diagnosis has been made, and the treatment well considered, do not be afraid to apply effective remedies. There is a great opportunity, not only for dealing with the question as it affects Glasgow, but also as it affects the whole of Scotland and England. The evil is deep, widespread, and increasing. The doctor's aspect of it is the least mischievous. The people themselves are injured, pauperised, degraded. It is of no significance that vapid twaddlers affect to sneer at the injury done to the well-to-do recipients of charity. These may be passed by as ignorant of the first principles of social economy and well-being

This is not a world of ease and play for the poor, any more than for the prosperous and the rich. It is a world of struggle and labour, and nobody can make it otherwise. The poor must struggle and strive for the victory, and like their wealthier neighbours they will be all the better for the struggle—better as men, better as citizens, better as competitors in the universal strife. By all means let the aged and the young, the blind and the maimed, and all of every degree who cannot help themselves, and who have no natural helpers, receive at the hand of charity the freest, the most bounteous, and the tenderest care. But let the strong man and the strong woman of every class stand up boldly and fight life's battle for themselves. He is no wise friend of man, no true philanthropist, no intelligent Christian, who robs any fellow-man of the noble sense of having won the battle of life by the courage of his own heart, and the strength of his own right hand. If the people of Glasgow can untie or cut this terrible Gordian knot, they will deserve well both of hospitals and the country at large.

